# Clover Root Exudates Favor *Novosphingobium* sp. HR1a Establishment in the Rhizosphere and Promote Phenanthrene Rhizoremediation

**DOI:** 10.1128/mSphere.00412-21

**Published:** 2021-08-11

**Authors:** Lázaro Molina, Zulema Udaondo, María Montero-Curiel, Regina-Michaela Wittich, Alicia García-Puente, Ana Segura

**Affiliations:** a Environmental Protection Department, Estación Experimental del Zaidín, Consejo Superior de Investigaciones Científicas, Granada, Spain; b Department of Biomedical Informatics, University of Arkansas for Medical Sciences, Little Rock, Arkansas, USA; University of Iowa

**Keywords:** clover exudates, phenomics, metabolomics, gene expression, rhizoremediation, polycyclic aromatic hydrocarbons (PAHs)

## Abstract

Rhizoremediation is based on the ability of microorganisms to metabolize nutrients from plant root exudates and, thereby, to cometabolize or even mineralize toxic environmental contaminants. *Novosphingobium* sp. HR1a is a bacterial strain able to degrade a wide variety of polycyclic aromatic hydrocarbons (PAHs). Here, we have demonstrated that the number of CFU in microcosms vegetated with clover was almost 2 orders of magnitude higher than that in nonvegetated microcosms or microcosms vegetated with rye-grass or grass. Strain HR1a was able to eliminate 92% of the phenanthrene in the microcosms with clover after 9 days. We have studied the molecular basis of the interaction between strain HR1a and clover by phenomic, metabolomic, and transcriptomic analyses. By measuring the relative concentrations of several metabolites exudated by clover both in the presence and in the absence of the bacteria, we identified some compounds that were probably consumed in the rhizosphere; the transcriptomic analyses confirmed the expression of genes involved in the catabolism of these compounds. By using a transcriptional fusion of the green fluorescent protein (GFP) to the promoter of the gene encoding the dioxygenase involved in the degradation of PAHs, we have demonstrated that this gene is induced at higher levels in clover microcosms than in nonvegetated microcosms. Therefore, the positive interaction between clover and *Novosphingobium* sp. HR1a during rhizoremediation is a result of the bacterial utilization of different carbon and nitrogen sources released during seedling development and the capacity of clover exudates to induce the PAH degradation pathway.

**IMPORTANCE** The success of an eco-friendly and cost-effective strategy for soil decontamination is conditioned by the understanding of the ecology of plant-microorganism interactions. Although many studies have been published about the bacterial metabolic capacities in the rhizosphere and about rhizoremediation of contaminants, there are fewer studies dealing with the integration of bacterial metabolic capacities in the rhizosphere during PAH bioremediation, and some aspects still remain controversial. Some authors have postulated that the presence of easily metabolizable carbon sources in root exudates might repress the expression of genes required for contaminant degradation, while others found that specific rhizosphere compounds can induce such genes. *Novosphingobium* sp. HR1a, which is our model organism, has two characteristics desirable in bacteria for use in remediation: its ubiquity and the capacity to degrade a wide variety of contaminants. We have demonstrated that this bacterium consumes several rhizospheric compounds without repression of the genes required for the mineralization of PAHs. In fact, some compounds even induced their expression.

## INTRODUCTION

Polycyclic aromatic hydrocarbons (PAHs) are among the most abundant contaminants in the environment. Several of these PAHs have been listed as priority pollutants to be eliminated from water, soil, or sediments because of their toxicity and/or their carcinogenic activity (1–4).

Bioremediation, the utilization of living organisms for the elimination of pollutants, is an environmentally friendly strategy that is considered to be more cost-effective than physicochemical treatments (5, 6). Indigenous microbial populations are capable of acting as bioremediation agents; however, this process, which is called natural attenuation, is generally very slow. To improve this process, bioaugmentation, which is the addition of microbes that provide catabolic functions for the degradation of these contaminants, can be used (7, 8).

Understanding the compatibility among the nonnative microorganism(s) to be inoculated and many other environmental factors (such as the type of soil, indigenous microbiota, nutrients, temperature, oxygen availability, and others) is important for achieving the desired outcome (9, 10). Bioaugmentation failures have been frequently attributed to the poor capacity of introduced species to use the scarce nutritional resources of soil in comparison with that of indigenous, well-adapted bacteria (11–13). It has been demonstrated that the rhizosphere is able to provide the resources necessary for bacterial nutrition through root exudation, favoring the proliferation of microbes and their metabolic activity (14–17). In addition, root exudates induce changes in the physicochemical properties of soils, influencing the processes of sorption and desorption. Specifically, low-molecular-weight organic acids released by plants have been reported to enhance the degradation of contaminants by different mechanisms: increasing phosphorus supply, providing easily degradable sources of carbon and energy, and/or enhancing the bioavailability of contaminants (18–21). In some cases, it has been shown that exudates induce specific degradation genes (22, 23).

Sphingomonads are well-known degraders of many contaminants, including PAHs, dibenzofurans or dioxins, halogenated phenols, pesticides, pharmaceuticals, and others, and are considered key players in the natural attenuation of the concentration of contaminants (24–30). Sphingomonads have been isolated from many different environments (26, 30–35) and are present in the rhizosphere, the phyllosphere, and the endosphere (26, 31–34, 36). Because of their ubiquity and biodegradative properties, they are considered good candidates for use in bioremediation strategies (37). Furthermore, several reports have indicated that sphingomonads improve plant growth under stressful conditions (26, 36, 38). However, currently there is very little information available about the molecular interactions of sphingomonads with the plant environment hosting them.

In rhizoremediation strategies, the selection of an appropriate plant is as important as the selection of a good biodegrading microbe. The selected plant species should be able to establish beneficial associations with the bacteria of interest, adapt to different climate conditions, and tolerate relatively high concentrations of the contaminant. Forage crops are ideal choices because they have well-established cultural practices and many are adapted to different climates. Herbaceous plants such as clover, grass, or legumes have been successfully explored for the elimination of total petroleum hydrocarbons or PAHs (39–42).

In this study, we have used the bacterial strain *Novosphingobium* sp. HR1a, isolated from plant rhizosphere, which is able to metabolize naphthalene, phenanthrene, and several high-molecular-weight PAHs (34). The molecular bases of PAH degradation are known, and the genes encoding the dioxygenase involved in the first step of PAHs degradation (*pahAB*) have been identified. The expression of these genes is controlled by the PahR regulator, and they are induced by different PAHs and by salicylate, an intermediate of their degradation pathway (34), which is also a key compound in plant signaling (43, 44). After analyzing the ability of strain HR1a to eliminate phenanthrene from microcosms vegetated with different herbaceous plants, we have been able to demonstrate that the HR1a strain is able to establish positive interactions with clover (Trifolium repens) and that these interactions are translated into improving the elimination of the contaminant (phenanthrene as model compound) in artificial microcosms. By metabolomic and transcriptomic analyses, we have studied the bacterial metabolism in the clover rhizosphere and we have demonstrated that clover exudates are used as carbon and nitrogen sources by *Novosphingobium* sp. HR1a, with the sugars, amino acids, and organic acids being consumed in sterilized systems in which only plants and the HR1a strain are growing (gnotobiotic systems). We have also demonstrated that the expression of *pahAB* genes (which encode the dioxygenase responsible for the initial step of the degradation of PAHs) is induced by clover exudates. Our results have unveiled the mechanisms by which this bacterium is able to become established in the clover rhizosphere, and we suggest that *Novosphingobium* sp. HR1a is an excellent option to use in rhizoremediation strategies.

## RESULTS

### *Novosphingobium* sp. HR1a is able to persist and to metabolize phenanthrene in nonsterile vegetated pots.

**FIG 1:**
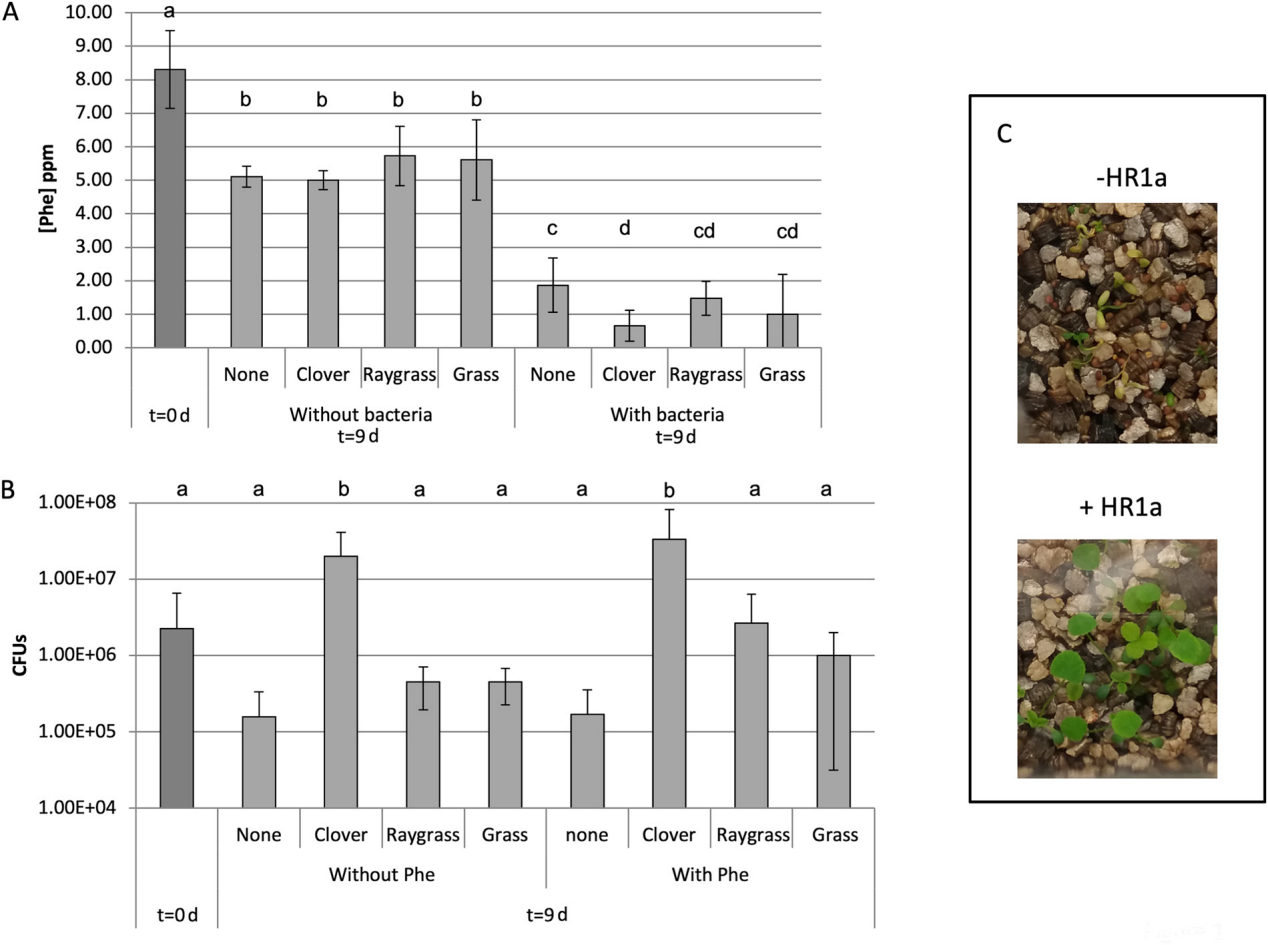
Phenanthrene degradation and growth of plant and *Novosphingobium* sp. HR1a in nonsterile microcosms. (A) Phenanthrene depletion in the microcosms in the presence and absence of bacteria (*Novosphingobium* sp. HR1a) and in the presence/absence of different plants. (B) Number of CFU in microcosms inoculated with *Novosphingobium* sp. HR1a at inoculation time (*t* = 0 d) and after 9 days (*t* = 9 d). (C) Growth of clover seedlings in pots contaminated with 1 mg of phenanthrene in the presence and absence of *Novosphingobium* sp. HR1a. Seeds and bacteria were added to the pots at the same time, and photographs were taken 6 days after planting. The different letters indicate that the treatments were statistically different at *P* < 0.05.

To select the best plant-bacteria combination for the elimination of phenanthrene, we set up nonsterile microcosms, and 10 ppm of phenanthrene was added to each pot at the beginning of the experiment. After 9 days, approximately 60 to 69% of the solvent-extractable phenanthrene remained in the pots, regardless of the presence or absence of any of the three plant species assayed (Fig. 1A). However, in pots inoculated with strain HR1a, phenanthrene decreased to 8 to 24% of the extractable amount. The elimination of phenanthrene was maximal in pots inoculated with clover plants (Fig. 1A). Accordingly, in pots with clover inoculated with *Novosphingobium* sp. HR1a, the number of CFU was almost 2 orders of magnitude higher than that in nonvegetated pots and significantly higher than that in the other microcosms (Fig. 1B), both in the presence and absence of phenanthrene. These results suggest that clover was able to secrete compounds that stimulate the proliferation of *Novosphingobium* sp. HR1a, thus improving phenanthrene degradation. In addition, it became obvious that the plants clearly benefited from the presence of the additionally augmented bacteria in contaminated microcosms by improved growth (Fig. 1C). Therefore, we selected clover plants for further studies.

### Utilization of clover exudates as carbon and nitrogen sources by *Novosphingobium* sp. HR1a.

**FIG 2:**
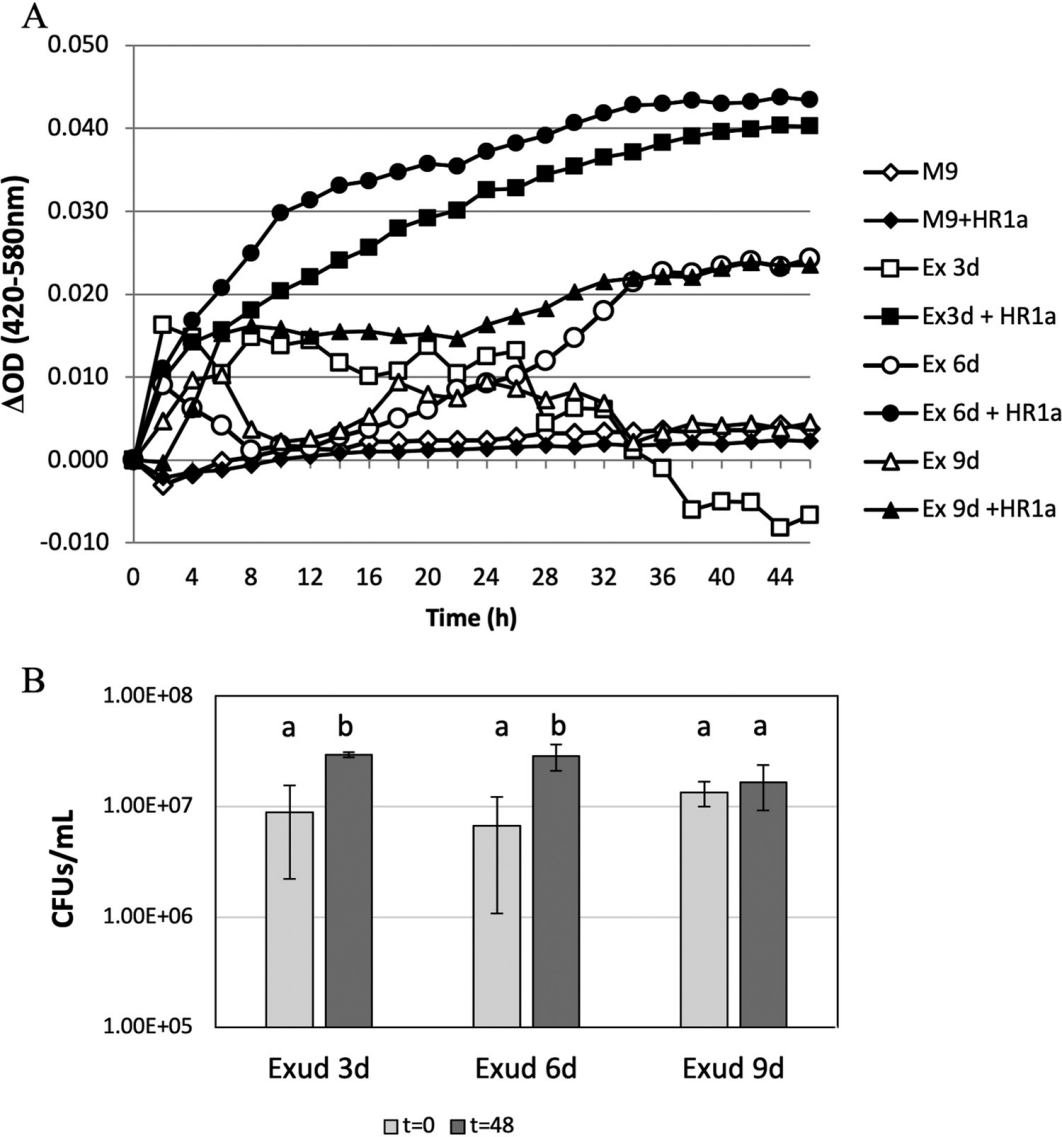
The growth of *Novosphingobium* sp. HR1a using the solution obtained from gnotobiotic systems as the sole nutrient source after 3, 6, and 9 days of plant growth. (A) Representative growth of *Novosphingobium* sp. HR1a in 96-well microplates using clover exudates obtained 3, 6, and 9 days after planting. The empty symbols are controls without inoculation, the black symbols are the cultures inoculated with *Novosphingobium* sp. HR1a, the squares are 3-day exudates, the circles are 6-day exudates, the triangles are 9-day exudates, and the diamonds are the controls in minimal M9 medium without carbon source. For clarity, in the graph, symbols represent time points for every 2 h. (B) Numbers of CFU at time zero and after 48 h of cultivation in clover exudates obtained after 3, 6, and 9 days. The bars represent the mean of three independent experiments ± standard error. The different letters indicate that there were statistical differences between the two time points at *P* < 0.05.

To demonstrate that clover root exudates are a good source of nutrients for *Novosphingobium* sp. HR1a, we produced clover exudates under sterile conditions and used them as the sole nutrient source for the growth of *Novosphingobium* sp. HR1a. Exudates obtained 3 and 6 days after planting were more efficient than those obtained after 9 days in the promotion of the growth of strain HR1a (Fig. 2A). Although the increments in optical density at 420 to 580 nm (OD_420–580_) were low (0.043 and 0.039 in exudates of 3 and 6 days, respectively), it should be noticed that the increment in OD_420–580_ under similar growth conditions but in M9 minimal medium with 2.5 mM glucose as the sole carbon source was 0.133 (not shown). Accordingly, the numbers of CFU ml^−1^ in the culture with exudates obtained after 9 days of incubation were not significantly different from those at the beginning of the experiment after 48 h of incubation. However, in the cultures grown with exudates obtained at 3 and 6 days, there were significant differences between the initial numbers of cells and the cell numbers in exudates obtained after 48 h (Fig. 2B). Therefore, as the seedling exudates obtained after 3 and 6 days of clover growth were the most effective for growing *Novosphingobium* sp. HR1a, only these were used in our subsequent experiments.

### Composition of clover root exudates obtained in the presence and absence of *Novosphingobium* sp. HR1a.

The gas chromatography-mass spectrometry (GC-MS) analyses of clover root exudates allowed us to identify 50 compounds (Table S1). The individual composition of the exudates was similar at the two different time points analyzed. However, in the exudates obtained after growing strain HR1a and clover together, there was a significant decrease in the percentage of sugars, organic acids, and amino acids, especially at day three, and a clear increase in the percentage of the catabolic intermediates of several amino acids (Table 1). When phenanthrene was added to the gnotobiotic systems, the proportions in the composition of root exudates did not significantly change in the absence of bacteria; however, in the presence of bacteria, there was a significant decrease in the proportion of amino acids on days 3 and 6 of the experiment, and an increase in the proportions of sugars on day 3, compared with the microcosm in the absence of the contaminant (Table 1; Table S1).

**TABLE 1:** Percentage of groups of compounds in clover exudates obtained from gnotobiotic systems planted with clover seeds after three (3D) and 6 days (6D)^*a*^

Gnotobiotic system	Sugars	Polyols	Amino acids	Amino acid inter.	Organic acids	Others
C 3D	30.23	22.61	13.62	3.65	27.27	2.62
C+H 3D	20.63	23.50	7.01	24.58	18.41	5.87
C+Phe 3D	28.28	24.29	14.26	3.19	27.25	2.74
C+H+Phe 3D	28.37	22.33	1.87	28.17	15.34	3.91
C 6D	26.85	28.12	6.41	3.66	29.05	5.91
C+H 6D	22.88	22.01	3.82	21.78	21.95	7.55
C+Phe 6D	26.52	29.32	7.49	3.82	28.02	4.82
C+H+Phe 6D	20.74	24.91	2.21	25.87	17.78	8.49

a C, clover; C+H, clover plus *Novosphingobium* sp. HR1a; C+Phe, clover plus phenanthrene; C+H+Phe, clover plus *Novosphingobium* sp. HR1a plus phenanthrene.

10.1128/mSphere.00412-21.3 TABLE S1 Relative quantities of the compounds identified in exudates from gnotobiotic systems at day 3 (3D) and day 6 (6D) after planting. C, clover; H, *Novosphingobium* sp. HR1a; Phe, phenanthrene; SD, standard deviation. Download Table S1, CSV file, 0.01 MB. Copyright © 2021 Molina et al. 2021 Molina et al. https://creativecommons.org/licenses/by/4.0/ This content is distributed under the terms of the Creative Commons Attribution 4.0 International license.

**FIG 3:**
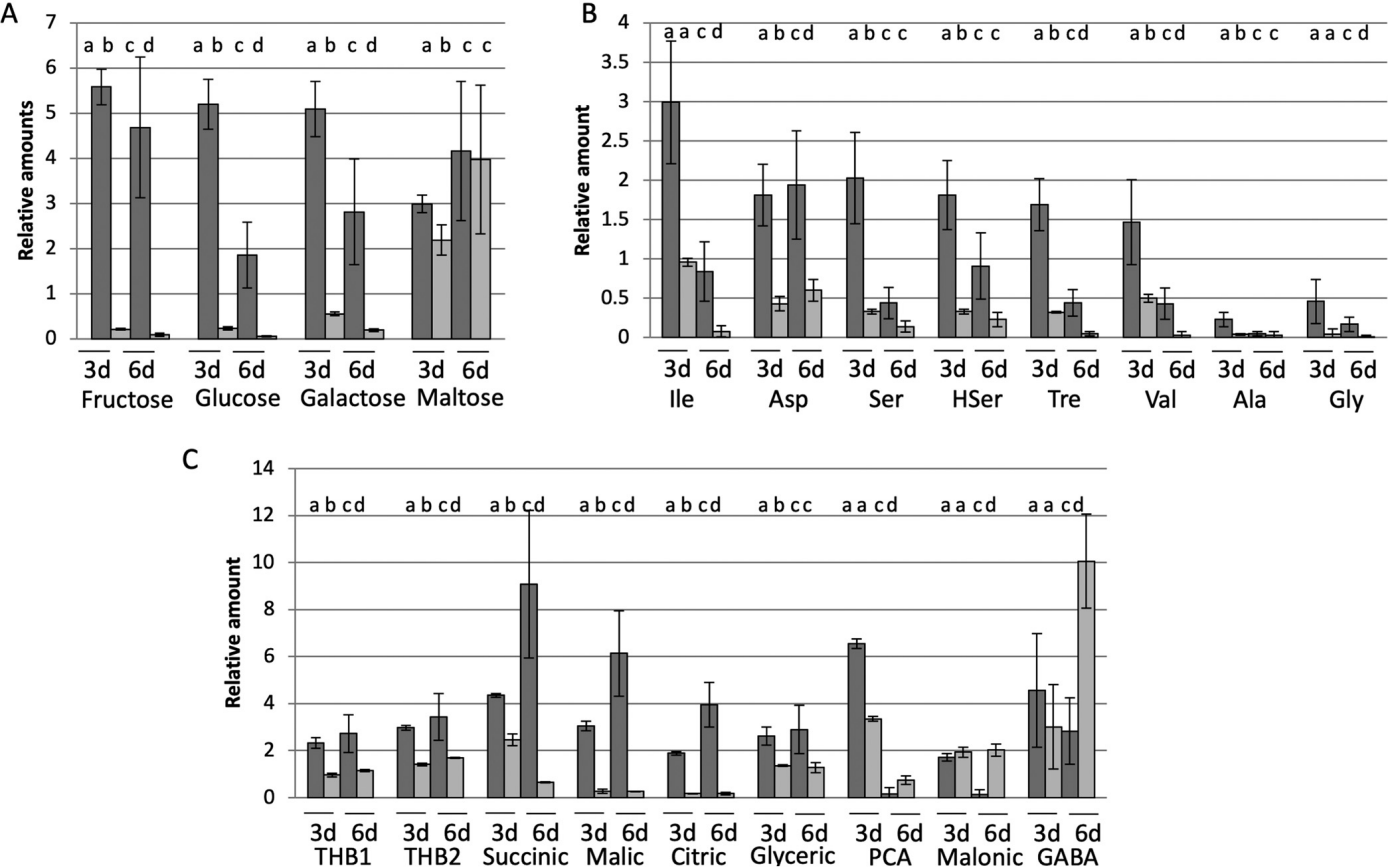
Relative amounts of (A) sugars, (B) amino acids, and (C) organic acids in noninoculated (dark gray) and inoculated (light gray) clover seedling exudates. The bars represent the mean of three independent measurements ± standard error; the different letters indicate statistical differences between the two time points and between the treatments at *P* < 0.05. Ile, isoleucine; Asp, aspartic acid; Ser, serine; HSer, homoserine; Tre, threonine; Val, valine; Ala, alanine; Gly, glycine; THB, 2,3,4-trihydroxybutyric acid (isomers 1 and 2 each); PCA, protocatechuic acid; GABA, γ-amino butyric acid.

**FIG 4:**
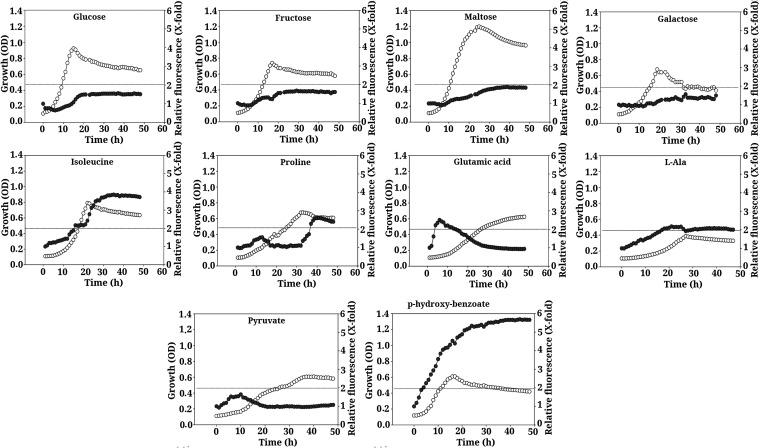
Growth course (empty symbols) and expression of P*_phaAB_* (solid symbols) during the growth of *Novosphingobium* sp. HR1a in M9 minimal medium with different compounds as the sole carbon source (sugars, amino acids, and organic acids). Growth was measured as OD_660_, and P*_pahAB_* expression was measured as the relative fluorescence (fluorescence normalized with OD_660_), as indicated in Materials and Methods. The dotted line indicates a 2-fold induction of fluorescence. The graph represents the mean of 6 experiments.

The most abundant sugars in clover exudates were saccharose and ribose (Table 2), but their concentrations were similar in exudates produced in the presence and absence of the bacteria, suggesting that either they were produced at a higher rate than the bacterial utilization rate or they were not at all being consumed by *Novosphingobium* sp. HR1a. To discriminate between these two possibilities, we tested the growth of strain HR1a in M9 minimal medium with saccharose or ribose as the sole carbon source. No growth was detected in any of the cases (not shown), confirming that these two sugars were not being consumed by our strain in the rhizosphere. Fructose, galactose, and glucose were present at lower concentrations in exudates obtained in the presence of bacteria than in bacterial-free exudates, suggesting that they were being consumed by strain HR1a in the gnotobiotic systems. Maltose was present at significantly lower concentrations in exudates from inoculated microcosms on day 3 but not on day 6 (Table 2; Fig. 3A). Accordingly, strain HR1a grew in mineral medium M9 when any of these four sugars was added as the sole carbon source (Fig. 4; Fig. S1A).

**TABLE 2:** Relative quantities of the compounds identified in gnotobiotic systems at day 3 (3D) and day 6 (6D) after planting, with clover (C) and clover with *Novosphingobium* sp. HR1a (C+H)

Identified compound	C 3D	C+H 3D	C 6D	C+H 6D
Sugars				
Saccharose	12.91 ± 0.43	15.47 ± 0.48	6.56 ± 2.28	5.66 ± 1.22
Ribose	10.03 ± 1.37	8.45 ± 0.30	17.12 ± 6.54	20.69 ± 0.98
Fructose	5.58 ± 0.40	0.21 ± 0.02	4.68 ± 1.56	0.09 ± 0.03
Glucose	5.20 ± 0.55	0.23 ± 0.04	1.86 ± 0.73	0.06 ± 0.01
Galactose	5.09 ± 0.61	0.55 ± 0.04	2.81 ± 1.17	0.19 ± 0.03
Maltose	2.99 ± 0.19	2.19 ± 0.34	4.16 ± 1.54	3.97 ± 1.65
Rhamnose	0.36 ± 0.01	0.30 ± 0.04	0.28 ± 0.06	0.25 ± 0.07
Polyols				
Myoinositol	10.66 ± 0.75	11.61 ± 0.40	14.31 ± 4.50	12.76 ± 2.51
d-Pinitol	12.55 ± 0.53	13.40 ± 0.53	15.07 ± 5.55	13.99 ± 1.50
Glycerol	8.32 ± 0.82	6.20 ± 0.09	9.85 ± 3.13	2.99 ± 0.89
Amino acids				
l-Ile	2.99 ± 0.78	0.96 ± 0.05	0.84 ± 0.38	0.08 ± 0.07
l-Glu	3.05 ± 0.82	2.95 ± 0.34	2.82 ± 1.01	3.46 ± 0.58
l-Pro	3.46 ± 0.73	3.41 ± 0.14	0.89 ± 0.34	0.53 ± 0.24
l-Asp	1.81 ± 0.39	0.43 ± 0.09	1.94 ± 0.69	0.60 ± 0.14
l-Ser	2.03 ± 0.58	0.33 ± 0.03	0.44 ± 0.20	0.14 ± 0.07
Homoserine	1.81 ± 0.44	0.33 ± 0.03	0.91 ± 0.42	0.23 ± 0.09
l-Tre	1.69 ± 0.33	0.32 ± 0.01	0.44 ± 0.17	0.05 ± 0.03
l-Val	1.47 ± 0.54	0.50 ± 0.05	0.43 ± 0.20	0.03 ± 0.05
l-Ala	0.23 ± 0.09	0.04 ± 0.01	0.05 ± 0.03	0.03 ± 0.05
l-Gly	0.46 ± 0.28	0.04 ± 0.07	0.17 ± 0.09	0.01 ± 0.02
Amino acid intermediates				
Oxoproline	3.96 ± 0.59	21.58 ± 1.86	4.22 ± 1.65	20.99 ± 2.87
2-Hydroxyglutaric	0.94 ± 0.01	1.87 ± 0.04	0.89 ± 0.25	1.35 ± 0.05
Methylmalonic	0.18 ± 0.17	1.91 ± 0.08	0.00 ± 0.00	0.79 ± 0.30
2-Aminoadipic	0.00 ± 0.00	1.51 ± 0.06	0.00 ± 0.00	1.38 ± 0.39
l-Indole-3-lactic acid	0.00 ± 0.00	5.18 ± 0.34	0.00 ± 0.00	3.38 ± 0.19
Organic acids				
Protocatechuic acid	6.55 ± 0.20	3.35 ± 0.10	0.16 ± 0.27	0.75 ± 0.18
2,3,4 Trihydroxybutyric (isomer 1)	2.32 ± 0.22	0.96 ± 0.08	2.72 ± 0.81	1.15 ± 0.04
2,3,4 Trihydroxybutyric (isomer 2)	2.97 ± 0.10	1.41 ± 0.05	3.43 ± 0.99	1.69 ± 0.02
Succinic acid	4.35 ± 0.08	2.46 ± 0.24	9.07 ± 3.14	0.65 ± 0.02
Malic acid	3.04 ± 0.20	0.26 ± 0.09	6.13 ± 1.81	0.26 ± 0.02
Glyceric acid	2.62 ± 0.38	1.36 ± 0.04	2.90 ± 1.02	1.28 ± 0.21
4-hydroxybutanoic (GABA)	4.56 ± 2.42	3.01 ± 1.79	2.83 ± 1.41	10.05 ± 2.00
Citric acid	1.89 ± 0.08	0.17 ± 0.01	3.95 ± 0.94	0.17 ± 0.06
Terephthalic acid	1.66 ± 0.06	2.42 ± 0.09	0.00 ± 0.00	0.00 ± 0.00
Malonic acid (propanedioic)	1.72 ± 0.16	1.93 ± 0.21	0.14 ± 0.20	2.02 ± 0.27
Glycolic acid	0.89 ± 0.04	0.51 ± 0.04	1.62 ± 0.73	2.66 ± 3.03
Gluconic acid (isomer 1)	0.40 ± 0.03	0.34 ± 0.03	0.54 ± 0.14	0.35 ± 0.09
Gluconic acid (isomer 2)	0.43 ± 0.04	0.21 ± 0.01	0.37 ± 0.00	0.22 ± 0.06
4-Hydroxybenzoic acid	0.81 ± 0.03	0.48 ± 0.05	1.02 ± 0.37	0.37 ± 0.04
Shikimic acid	0.71 ± 0.04	0.49 ± 0.07	0.23 ± 0.07	1.03 ± 0.08
Deoxytetronic acid	0.57 ± 0.01	0.35 ± 0.06	0.76 ± 0.25	0.24 ± 0.03
Oxalic acid	0.58 ± 0.05	0.39 ± 0.03	1.24 ± 0.45	0.36 ± 0.05
Glutaric acid (pentanedioic)	0.35 ± 0.07	1.50 ± 0.17	0.79 ± 0.34	1.04 ± 0.17
2-Butenedioic	0.34 ± 0.08	0.15 ± 0.01	0.63 ± 0.27	0.13 ± 0.03
Azelaic acid	0.28 ± 0.03	0.35 ± 0.03	0.41 ± 0.15	0.39 ± 0.03
Lactic acid	0.20 ± 0.04	1.18 ± 0.13	0.38 ± 0.16	3.29 ± 3.91
Galactaric acid	0.23 ± 0.05	0.20 ± 0.06	0.33 ± 0.10	0.36 ± 0.17
Tartaric acid	0.17 ± 0.01	0.14 ± 0.01	0.20 ± 0.08	0.13 ± 0.01
Ethylmalonic/methylsuccinic acid	0.16 ± 0.01	0.18 ± 0.03	0.49 ± 0.25	0.36 ± 0.08
Aconitic acid	0.05 ± 0.09	0.07 ± 0.12	0.13 ± 0.22	0.00 ± 0.00
Others				
Urea	2.70 ± 0.20	2.53 ± 0.18	6.81 ± 2.16	4.09 ± 1.13
*N*-Methacryloylglycine	0.52 ± 0.02	3.04 ± 0.23	0.57 ± 0.29	3.87 ± 1.31
Uracil	0.44 ± 0.05	2.22 ± 0.18	0.87 ± 0.35	2.24 ± 0.45

10.1128/mSphere.00412-21.1 FIG S1 *Novosphingobium* sp. HR1a growth in M9 minimal medium with (A) sugars (10 mM) as the sole carbon source or (B) amino acids (5 mM) as the sole carbon source. LB-grown overnight cultures of *Novosphingobium* sp. HR1a (OD_660_ ≈ 2.5) were washed three times with distilled water and diluted into the M9 minimal medium to reach an OD_660_ of 0.005. Two hundred microlitres of the M9 minimal medium with each carbon source was dispensed into wells of honeycomb microplates (OY Growth Curves Ab Ltd., Raisio, Finland). Wells inoculated with M9 minimal medium but without carbon sources were included in the experiment as controls. Three independent experiments with 8 replicas each were carried out. Growth was monitored using an FP-1100-C Bioscreen C MBR (type) analyzer system (OY Growth Curves Ab Ltd., Raisio, Finland) at 30°C with continuous agitation. Turbidity was measured using a sideband filter at 420 to 580 nm every 60 minutes for 2 days. Download FIG S1, TIF file, 0.4 MB. Copyright © 2021 Molina et al. 2021 Molina et al. https://creativecommons.org/licenses/by/4.0/ This content is distributed under the terms of the Creative Commons Attribution 4.0 International license.

The concentration of most of the detected amino acids, except glutamic acid and proline, decreased in the presence of bacteria (Table 2; Fig. 3B). However, *Novosphingobium* sp. HR1a was able to grow in M9 mineral medium containing glutamic acid or proline as the sole carbon source (Fig. S1B). All the intermediates of amino acid metabolism identified in this study increased their concentration in the presence of bacteria, with the increase in oxoproline being especially significant (Table 2).

Among the most abundant organic acids in clover exudates (Table 2), succinic, malic, 2,3,4-trihidroxybutyric, citric, and glyceric acids were apparently consumed by *Novosphingobium* sp. HR1a in the clover rhizosphere (Fig. 3C), while protocatechuic acid, malonic acid, and γ-aminobutyric acid (GABA) accumulated in the presence of bacteria after 6 days (Fig. 3C). Terephthalic acid, which was relatively abundant in the exudates obtained after 3 days of planting/inoculation (1 to 2%), was not detected in exudates obtained after 6 days (Table 2) either in the presence or in the absence of the bacteria. Other organic acids, which were consumed by strain HR1a, are gluconic (isomer 2), 4-hydroxybenzoic, deoxytetronic, oxalic, and 2-butenedioic acid (Table 2). The shikimic acid concentration decreased in the presence of our bacterium in the exudates of day 3, but its concentration was found increased on day 6 (Table 2).

Compounds such as uracil or *N*-methacryloylglycine seemed to have accumulated in the rhizosphere of clover plants as a consequence of the presence of strain HR1a, as they were detected in higher quantities in exudates with bacteria than in exudates without strain HR1a (Table 2).

In the gnotobiotic systems to which phenanthrene had been added, phenanthrene quantities in the absence of our bacteria were higher than in their presence. Salicylate, which is an intermediate in the pathway of phenanthrene degradation, was 6.4-fold and 6.2-fold more abundant in the exudates of 3 and 6 days, respectively, in the presence of HR1a than in the exudates without added bacteria (Table S2).

10.1128/mSphere.00412-21.4 TABLE S2 Identification of phenanthrene and salicylate in exudates. The relative amounts of each compound were calculated by dividing the peak area between the milligrams of lyophilized exudate used in the analysis. C, clover; Phe, phenanthrene; 3D, exudates obtained 3 days after inoculation; 6D, exudates obtained after 6 days. Download Table S2, DOCX file, 0.01 MB. Copyright © 2021 Molina et al. 2021 Molina et al. https://creativecommons.org/licenses/by/4.0/ This content is distributed under the terms of the Creative Commons Attribution 4.0 International license.

### *pahAB* genes are expressed in the presence of clover exudates.

The presence of carbon sources other than PAHs in the rhizosphere could produce so-called catabolic repression, under which the microorganisms preferentially consume more easily mineralizable carbon sources, affecting the expression of the contaminant-degradation genes (45). Therefore, we analyzed the expression of the *pahAB* genes encoding the initial dioxygenase responsible for phenanthrene degradation in *Novosphingobium* sp. HR1a by using the reporter strain *Novosphingobium* sp. HR1a (pKSPA-1), which carries the P*_pahA_*-green fluorescent protein (GFP) construct (34), while growing it in M9 minimal medium with different carbon sources.

l-Isoleucine (expression level, 4-fold), l-proline (2.5-fold), l-alanine (2-fold), glutamic acid (2.5-fold), and *p*-hydroxybenzoate (5.5-fold) were able to induce the expression of the *pahAB* genes (Fig. 4). Furthermore, the expression of these genes did not decrease while the strain was growing with any of the sugars tested (glucose, fructose, maltose, or galactose) or with pyruvate (Fig. 4), indicating that the basal expression from the *pahAB* promoter was not repressed by the presence of these compounds.

**FIG 5:**
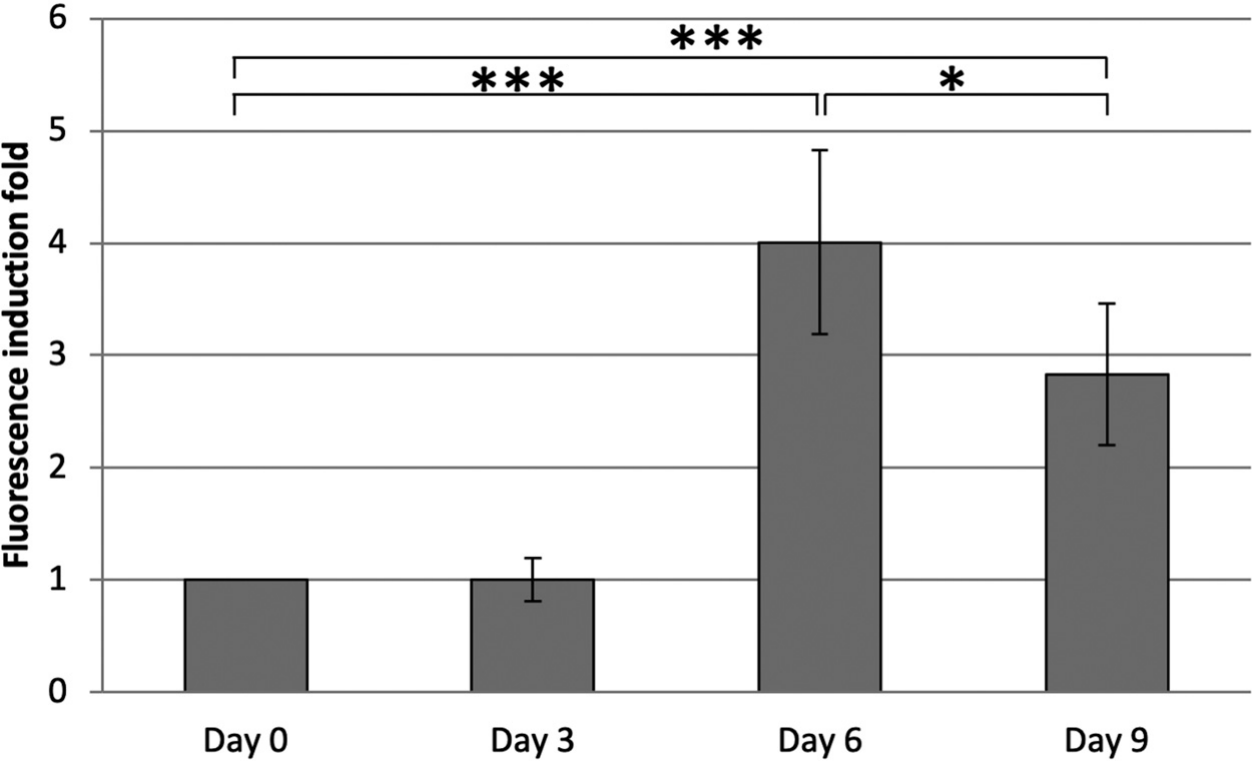
Increased GFP fluorescence with time in gnotobiotic systems with clover. The fluorescence measurements were normalized to the number of CFU in the pots. The induction-fold was calculated as the fluorescence per CFU at any time relative to the fluorescence per CFU at day 0. Changes between days 0 and 3 were not statistically significant. ***, *P* = 0.002; *, *P* = 0.041.

To further explore the effect of rhizospheric exudates on the expression of the *pahAB* promoter *in vivo*, we inoculated *Novosphingobium* sp. HR1a (pKSPA-1) into gnotobiotic systems with clover seeds, and GFP fluorescence and the numbers of CFU were measured in the pots at days 0, 3, 6, and 9. Our results showed that there was a 4-fold induction of GFP fluorescence after 6 days (Fig. 5). This result suggests that, under our experimental conditions, the root exudates were able to induce the expression of the dioxygenase promoter.

### Genes expressed in the rhizosphere.

To further investigate the metabolic abilities of *Novosphingobium* sp. HR1a in the clover rhizosphere, we carried out a transcriptomic analysis to identify the genes that were expressed in the presence of clover roots and their respective exudates. In the gnotobiotic systems without clover seedlings, the number of CFU decreased by about 2 orders of magnitude from 1.63 × 10^5^ (±5.06 × 10^4^) at inoculation time to 1.43 × 10^3^ (± 4.08 × 10^2^) after 9 days. However, there was a significant increase in the number of CFU in the presence of clover seedlings, reaching 1.79 × 10^7^ ± 3.62 × 10^6^ CFU in this period of time. This result clearly indicates that the bacteria were actively growing in the systems. Accordingly, the level of expression in the gnotobiotic systems of well-known reference genes was relatively high (Table S3), thus supporting the active metabolic state of the cells.

10.1128/mSphere.00412-21.5 TABLE S3 Level of expression (FPKM) of reference genes in *Novosphingobium* sp. HR1a growing in the gnotobiotic systems. Download Table S3, DOCX file, 0.01 MB. Copyright © 2021 Molina et al. 2021 Molina et al. https://creativecommons.org/licenses/by/4.0/ This content is distributed under the terms of the Creative Commons Attribution 4.0 International license.

**FIG 6:**
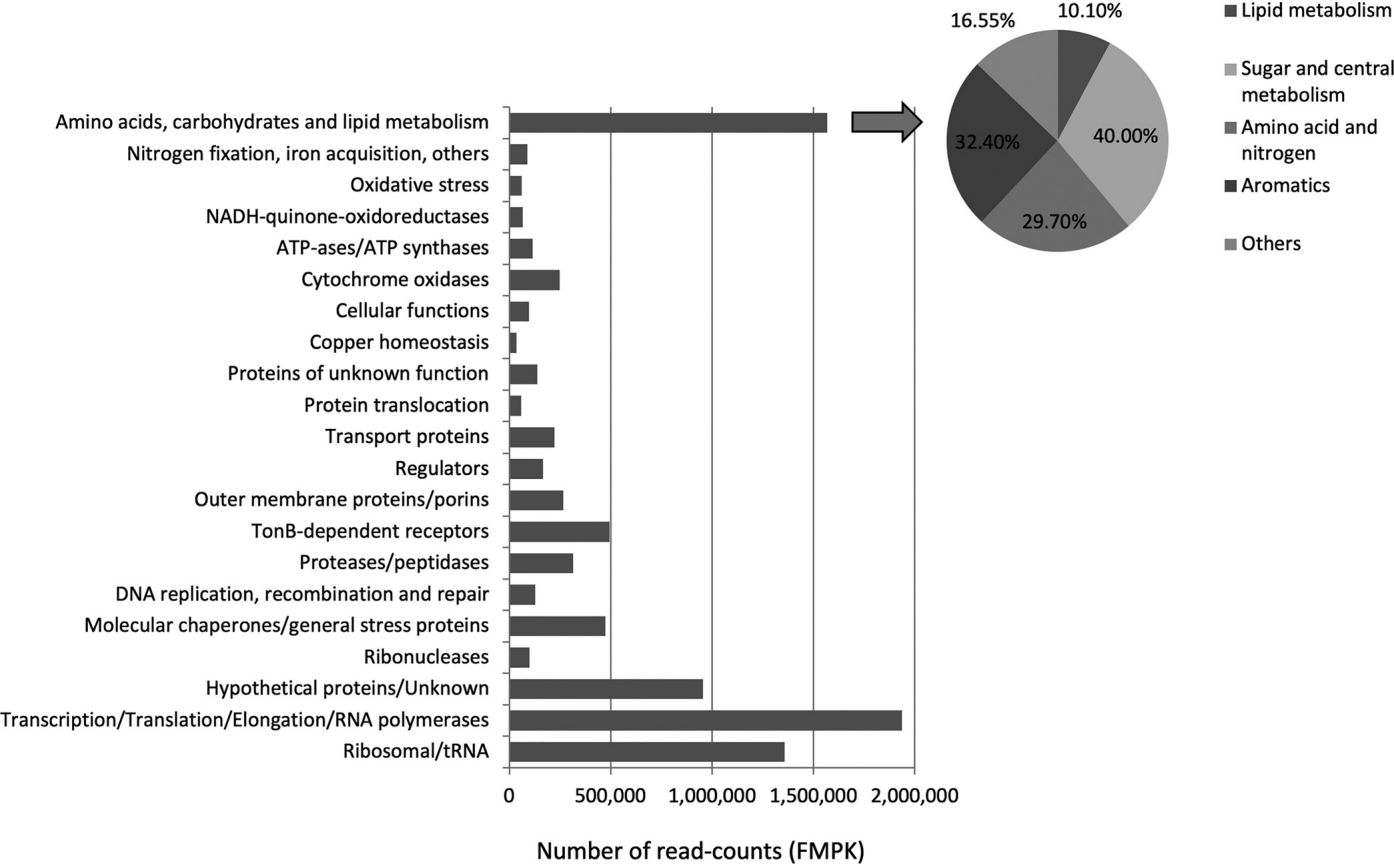
Functional analysis of the most abundant transcripts in the gnotobiotic systems (number of read counts as fragments per kilobase of transcript sequence per millions of sequenced base pairs [FMPK]) and relative abundance (percentage) of genes which are involved in lipid, sugar and central metabolism, amino acid and nitrogen metabolism, aromatic metabolism, and other metabolic processes.

We focused our analysis on the 416 genes whose levels of expression were higher than that of *recA*; expression of these genes in the gnotobiotic systems represented about 67% of the total read counts. The most abundant groups involved were ribosomal proteins, proteins involved in translation initiation, elongation, and transcription termination, RNA polymerases, hypothetical proteins, and genes related with metabolism in general (Fig. 6; Table S4). Among the latter group, the genes involved in sugar and central metabolism represented 40% of the read counts, followed by transcripts related with amino acid metabolism, ammonium, and urea cycles (Fig. 6). The genes involved in *p*-hydroxybenzoate and protocatechuate degradation were also being expressed in the clover rhizosphere (Fig. 6; Table S4).

10.1128/mSphere.00412-21.6 TABLE S4 Level of expression (FPKM) of *Novosphingobium* sp. HR1a genes growing in the clover rhizosphere (TH). Download Table S4, CSV file, 1.1 MB. Copyright © 2021 Molina et al. 2021 Molina et al. https://creativecommons.org/licenses/by/4.0/ This content is distributed under the terms of the Creative Commons Attribution 4.0 International license.

## DISCUSSION

In bioaugmentation strategies for contaminant bioremediation, the selected introduced bacteria have to establish interactions with a dynamic environment, and the balance between the cooperative and antagonistic forces will determine the outcome of this strategy (46). When bioaugmentation is required in rhizoremediation strategies, the biodegrading bacteria have to be metabolically active and have to be able to mineralize the contaminant. We have demonstrated that the coexistence of clover seedlings and bacterial cells of *Novosphingobium* sp. HR1a allows for the establishment of mutual beneficial interactions, in which the bacteria obtain carbon and nitrogen for growth from the plant while the plant benefits from the elimination of the contaminant. Although this observed beneficial effect has not been deeply explored in this study, the alleviation of the toxic effect of the contaminants toward plants by rhizospheric bacteria has already been demonstrated in other plant-bacteria systems (26, 47). Increasing plant growth and yield in contaminated soils will allow the utilization of marginal soils in agricultural production. The possible accumulation of contaminants or some intermediates in the aerial part of the plant will preclude the utilization of contaminated soils for food crops; however, utilization of crops for the generation of bio-energy will allow the increase of the economic revenues during bioremediation.

Plants exude around 11 to 40% of their photosynthetically accumulated carbon through root exudation (48), the primary metabolites being mainly composed of organic acids, amino acids, and sugars (49–51). The composition of root exudates varies with plant age, physiological conditions, climate, and plant species and in response to the presence of bacterial strains (50, 52, 53). Metabolites exuded by plant roots make a significant contribution to the shaping of their root microbiome (54). Although many studies have focused on the secretion of secondary metabolites as compounds responsible for specific plant-bacteria interactions (55, 56), other authors have suggested that the preference of certain bacteria for the consumption of specific compounds of the plant exudates could be a major driving force for shaping root microbiota (57, 58). Our studies have shown significant experimental evidence, indicating that sugars (at least glucose, galactose, and fructose), amino acids (at least valine, isoleucine, and tyrosine), and organic acids (at least citric, malic, succinic, *p*-hydroxybenzoate, and protocatechuic acid) are being consumed in the clover rhizosphere in the presence of *Novosphingobium* sp. HR1a: (i) the smaller amount of certain compounds in exudates obtained from gnotobiotic systems inoculated with strain HR1a compared with the amount of those from the noninoculated systems, (ii) the results obtained concerning the utilization of individual compounds as nitrogen or carbon sources by the bacteria, (iii) the accumulation of certain intermediates of the degradation pathway in the rhizosphere in the presence of the bacteria but not in their absence, (iv) the fact that the bacteria were able to grow *in vitro* using clover exudates as carbon and nitrogen sources, and (v) the results of the transcriptomic analysis of strain HR1a growing in the gnotobiotic systems.

Carbohydrates, proteins, and lipids have been reported as the major reserves in seeds (59). Our exudates were collected just after germination (3 days after planting) and at the very early growth phase of the clover (6 days after planting). Therefore, the level of sugars in this study (around 25% of the total compounds) was higher than has been reported in other studies with clover (51). It has been determined that the gene category “carbohydrate metabolism and transport” was expanded in the plant-associated organisms (60) and that the utilization of sugars may represent an adaptive mechanism in the rhizosphere that could be exploited as a selective advantage over other rhizobacteria.

In addition to constituting carbon sources, organic acids have an important role in the mobilization of contaminants during bioremediation (19, 20, 47, 61). The bioavailability of organic contaminants in soil decreases with time and varies among different types of soils. Furthermore, several studies have suggested that the bioavailability is reduced in those soils with more than 2% organic matter content (57, 62). However, we did not observe this effect; our experiments were conducted with vermiculite as the substrate, phenanthrene was added immediately before planting, and probably the phenanthrene was not immobilized onto the matrix. Other organic acids, such as malic, azelaic, γ-aminobutyric acids (GABA), and auxins, all of which were identified in our study, have been described as signaling molecules in the rhizosphere or of being involved in plant responses (52, 63, 64). The role, if any, of these compounds in the clover-*Novosphingobium* interaction is still unknown.

**FIG 7:**
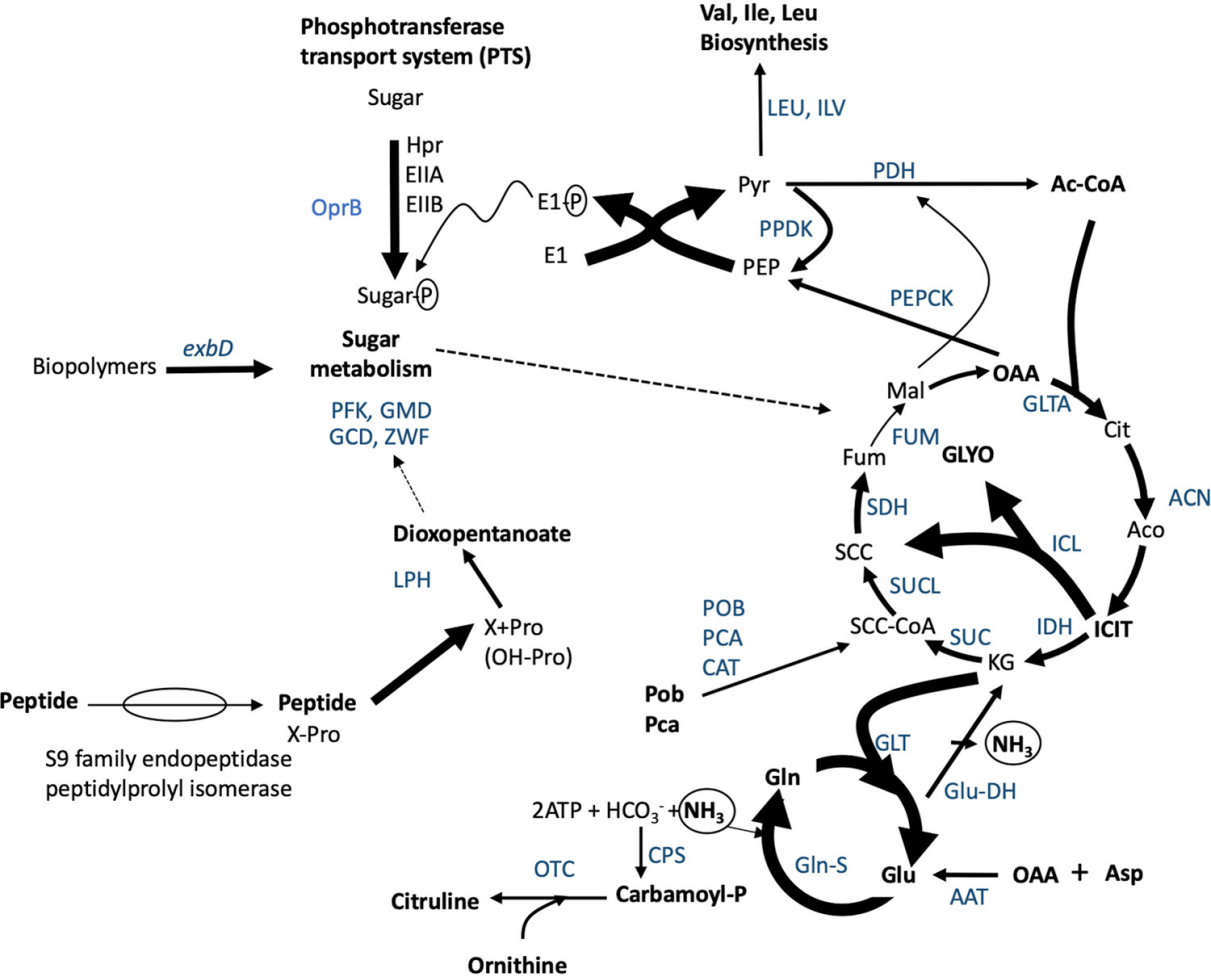
Schematic representation of the metabolic processes that are active in the gnotobiotic systems as could be deduced from gene expression levels. Wider arrows represent processes highly expressed in the gnotobiotic systems while thinner arrows correspond to low level of transcripts. The blue letters indicate enzymatic activities encoded by genes with high level of transcription; some of them represent several different genes (activities). *exbD*, biopolymer transport protein; PFK, fructose-1-6-bisphosphatase; GMD, GDP-mannose 4,6 dehydratase; GCD, glucose dehydrogenase (PQQ-dependent); ZWF, glucose-6-phosphate dehydrogenase; OprB, sugar porin; EI (EI-P, phosphorylated EI component), EIIA, EIIB, and HPr, components of the phosphotransferase transport system; PPDK, pyruvate phosphate dikinase; PEPCK, PEP carboxykinase; PDH, pyruvate dehydrogenase E1 and pyruvate dehydrogenase E2 component (dihydrolipoamide acetyltransferase); ACN, aconitate hydratase; IDH, isocitrate dehydrogenase; ICL, isocitrate lyase; SUC, 2-oxoglutarate dehydrogenase E1 component, 2-oxoglutarate dehydrogenase E2 component (dihydrolipoamide succinyltransferase); SUCL, succinyl-CoA-ligase; SDH, succinate dehydrogenase; FUM, fumarate hydratase; GLTA, citrate synthase; CPS, carbamoyl P synthetase; OTC, ornithine transcarbamoylase; Glu-DH, glutamate dehydrogenase; GLT, glutamate synthetase; Gln-S, glutamine synthetase; AAT, aspartate aminotransferase; LEU, 3-isopropyl malate dehydratase; ILV, acetolactate synthase (large subunit) acetolactate synthase (small subunit), acetohydroxy acid isomeroreductase, dihydroxy-acid dehydratase, branched-chain amino acid aminotransferase; LPH, hydroxy proline epimerase and 1-pyrroline-4-hydroxy-2-carboxylate deaminase; PCA, protocatechuate dioxygenase; POB, 4-hydroxybenzoate 3-monooxygenase; CAT carboxymuconolactone decarboxylase. Pyr, pyruvate; PEP, phosphoenol-pyruvate; Ac-CoA, acetyl-CoA; Cit, citrate; Aco, aconitate; ICIT, isocitrate; KG, α-ketoglutarate; SCC-CoA, succinyl-CoA; SCC, succinate; Fum, fumarate; Mal, malate; OAA, oxaloacetate; Val, l-valine; Ile, l-isoleucine; Leu, l-leucine; Asp, l-aspartate; Pob, *p-*hydroxy-benzoate; Pca, protocatechuate; Gln, l-glutamine; Glu, l-glutamate; X-Pro, peptide with an N-terminal proline.

Ammonium and amino acids have been reported as the major nitrogen compounds released by white clover (65). It was previously reported that glycine and serine were the major amino acids exuded in white clover, followed by alanine, glutamic acid, and aspartic acid (65, 66). Although we have identified all of these amino acids, in our study proline, glutamic acid, and isoleucine were the most abundant ones. In the gnotobiotic systems, *Novosphingobium* sp. HR1a genes for ammonium incorporation into the cell through the urea cycle (carbamoylphosphate synthase and ornithine carbamoyltransferase) were actively transcribed (Fig. 7; Table S4). The high expression of the glutamate synthetase and the glutamine synthetase, and the apparent lack of consumption of these amino acids in exudates, suggests that glutamine is being used as a nitrogen reservoir (Fig. 7). Furthermore, l-5-oxoproline (pyroglutamic acid), which accumulated in exudates in the presence of the HR1a strain, has been proposed as functioning as an analogue or reservoir of glutamate. However, proline and glutamate have also been recognized as compatible solutes (67, 68), so we can also hypothesize that the accumulation of these two compounds in the rhizosphere could be used to counteract the osmotic stress in the gnotobiotic systems. This osmotic stress was revealed by the high level of polyols detected in clover exudates, as glycerol and d-pinitol have an active role as osmoprotectants, while myoinositol is a precursor of d-pinitol in plant species (69, 70). It is, therefore, plausible that in our systems that were kept as simple as possible (crystal balls, water, and iron), plants and/or bacteria synthesized these compounds as a response to osmotic stress. Moreover, polyols, saccharose, and ribose can also contribute to the generation and responses of osmotic stress in our systems (71). The presence of compatible solutes in the rhizosphere and the high expression of several stress-related genes suggest that the HR1a strain is well equipped with resistance mechanisms which enable it to survive in harsh and stress conditions. It has been demonstrated that *Novosphingobium* sp. HR1a improves plant growth under saline stress (34, 72).

The results obtained in our study suggest that *Novosphingobium* sp. HR1a is metabolically active in the presence of clover exudates and that it is actively consuming compounds present in root exudates (Fig. 7). By using a reporter plasmid to analyze the expression of the *pahAB* genes, which encode the dioxygenase responsible for the initial degradation of several PAHs, we have demonstrated that these genes are expressed in the clover rhizosphere and are being induced by several rhizospheric compounds (*p*-hydroxybenzoic acid, l-isoleucine, l-alanine, and l-glutamic acid). As a consequence, phenanthrene is actively removed from the microcosm. We cannot exclude the possibility that, in addition to the active expression of the dioxygenase genes, the presence of other carbon sources increases the PAH degradation as a consequence of cometabolism in which the cells obtained the energy required for PAH degradation by the catabolism of root exudates (73).

The results obtained in this study have advanced our knowledge about the molecular basis of the interaction between *Novosphingobium* sp. HR1a and clover and suggest that *Novosphingobium* sp. HR1a is an excellent candidate to use in the rhizoremediation of PAH-contaminated soils.

## MATERIAL AND METHODS

### Nonsterile microcosm setup and CFU monitoring.

The microcosms consisted of 120-ml plastic pots containing a volume of 80 ml of vermiculite. A *Novosphingobium* sp. HR1a mutant, resistant to rifampin, was used in this study. The strain was grown overnight in M9 minimal medium (74) supplemented with 20 mM glucose and rifampin (10 μg ml^−1^) under sterile conditions, in an incubator at 30°C with continuous orbital shaking (200 rpm). The bacterial cultures were then diluted with nonsterile tap water to reach a final optical density at 600 nm (OD_660_) of 0.005. The initial viable cell numbers of *Novosphingobium* sp. HR1a were determined by spotting serial dilutions (10^−2^, 10^−4^, and 10^−6^) of the inoculated tap water on Luria Bertani (LB) solid medium supplemented with rifampin (10 μg ml^−1^) and cycloheximide (50 μg ml^−1^). The plates were incubated for 3 days at 30°C, and the numbers of CFU were counted.

Fifty milliliters of nonsterile tap water (control) or a bacterial solution was added to each microcosm to obtain a moisture content corresponding to 100% of the water holding capacity of the microcosms. Nonsterilized seeds of clover (Trifolium repens, 150 mg), rye-grass (Lolium perenne, 300 mg), or grass (rye-grass Engl. Pascal 30%, Festuca arundinacea Rendition 40%, and *Festuca arundinacea* Borneo 30%; 300 mg) were then added to cover the surface of the microcosms. The microcosms were placed inside a seed propagator in a growth chamber with day (24°C/16 h) and night (20°C/8 h) cycles.

Samples were taken 9 days after inoculation to analyze the numbers of CFU. Tap water was added to the microcosms to reach 100% water holding capacity prior to thoroughly mixing the microcosms with a spatula. Then, the number of CFU was determined by serial dilutions from microcosms on LB plates supplemented with rifampin (20 μg ml^−1^) and cycloheximide (50 μg ml^−1^).

### Phenanthrene extraction from nonsterile microcosms and phenanthrene quantification.

Phenanthrene (0.5 mg), from a 10 g liter^−1^ phenanthrene stock solution in acetone, was added to 40 ml of vermiculite placed in plastic pots. After thorough mixing with the vermiculite, the acetone was allowed to evaporate at room temperature overnight. The next day, another 40 ml of vermiculite was added to the plastic pots and thoroughly mixed. One hundred fifty milligrams of nonsurface sterilized clover seeds was added to each pot. The overnight-grown culture of *Novosphingobium* sp. HR1a (grown on M9 minimal medium plus glucose and rifampin as above) was diluted to an OD_660_ of 0.005 with nonsterile tap water. Seventy milliliters of this dilution was used to water the pots. Control pots were filled with 70 ml of nonsterile tap water. Immediately after watering, four pots were sacrificed to extract the phenanthrene (day 0). For this purpose, 30 ml of methanol was added to the pot and mixed thoroughly, and 1 ml of the resulting mixture was centrifuged to remove the debris and used for high-performance liquid chromatography (HPLC) analysis. For the phenanthrene analysis at the initial time points, 10-fold dilutions of the samples were used for analyses by HPLC.

The rest of the pots were cultivated in the plant chamber as described above. After 9 days, samples for counting the number of CFU by drop plating were taken (as described above), 30 ml of methanol was added, and the pots were treated as described above. Four independent parallel experiments with two replicas of each were carried out.

The phenanthrene concentrations were determined by HPLC as reported previously (26). The efficiency of the extraction procedure was 83% ± 11%.

### Gnotobiotic system setup.

The gnotobiotic systems consisted of glass jars with 50 ml of glass beads, 25 ml of sterile water with Fe-EDTA (2.5 mM), and 100 mg of surface-sterilized clover seeds. Seed surface sterilization was obtained by washing the seeds with 70% ethanol for 10 min, and then the seeds were thoroughly rinsed three times in sterile demineralized water, washed with 25% sodium hypochlorite solution for 10 min, and rinsed again three times with sterile demineralized water. The plants were grown for 3, 6, and 9 days (depending on the experiment) in a plant chamber at 24°C with a light cycle (103 to 279 μmol m^−2^s^−1^) of 16 h and at 20°C with a dark cycle of 8 h.

To obtain exudates after the growth of strain HR1a, we grew *Novosphingobium* sp. HR1a overnight in LB medium. The cells were centrifuged, washed three times with distilled sterile water, and used to inoculate the gnotobiotic systems to reach an initial OD_660_ of 0.1 at the same time that surface-sterilized clover seeds were added. The gnotobiotic systems were grown in the plant chamber as described above.

### Growth of HR1a with clover exudates.

To study the growth of *Novosphingobium* sp. HR1a using root exudates as the sole carbon and energy source, noninoculated gnotobiotic systems were prepared as described above, and at days 3, 6, and 9 after planting, the exudates were collected. LB-grown overnight cultures of *Novosphingobium* sp. HR1a (OD_660_ ≈ 2.5) were washed three times with distilled water and diluted into the exudates to reach an OD_660_ of 0.005. Two hundred microliters of the different inoculated and noninoculated exudates was dispensed into wells of honeycomb microplates (OY Growth Curves Ab Ltd., Raisio, Finland). The wells inoculated with M9 minimal medium but without carbon sources were included in the experiment. Three independent experiments with 8 replicas each were carried out. Growth was monitored using an FP-1100-C Bioscreen C MBR (type) analyzer system (OY Growth Curves Ab Ltd., Raisio, Finland) at 30°C with continuous agitation. Turbidity was measured using a sideband filter at 420 to 580 nm every 60 min for 2 days. Increments in turbidity represented the differences between the initial turbidity (time zero) and the turbidity at the different time points.

Two replicas of three independent experiments were used for CFU counting by drop plating (as described before). Samples were taken at the beginning of the experiment and after 48 h of incubation.

### Analysis of root exudates by gas chromatography-mass spectrometry.

Ten milliliters of the Fe-EDTA solution, obtained from 4 independent gnotobiotic systems, containing exudates inoculated or not with *Novosphingobium* sp. HR1a were collected after 3 and 6 days. Then, 4 × 10 ml of each solution with exudate was mixed, freeze-dried, and stored at −80°C.

Two to three milligrams of lyophilized exudates were resuspended in 100 μl methanol containing 0.2 mg × ml^−1^ ribitol as an internal standard. The solution was dried under nitrogen and derivatized by the addition of 60 μl of pyridine containing 20 mg ml^−1^ of methoxyamine. After vortexing for 10 s, the resulting solution was incubated for 120 min at 70°C. After cooling and a brief centrifugation at 1,000 × *g*, 100 μl of *N*,*O*-bis(trimethylsilyl)trifluoroacetamide with 1% (vol/vol) of the silylation agent trimethylchlorosilane was added and the resulting solution was incubated at 70°C for 30 min. Once cooled to room temperature and after another brief centrifugation, 100-μl aliquots of the samples were transferred to chromatographic vials. Then, 1-μl samples were injected at 230°C into a 450 GC-240 MS Varian gas chromatograph-mass spectrometer using split mode (1:20). The helium carrier gas flow was 1 ml min^−1^, and the J&W DB-5 MS UI 30 mm × 0.25 mm × 0.25 μm capillary chromatography column (Agilent Technologies) was used. The oven ramp was set as follows: 70°C (5 min) to 245°C at 5°C/min to 310°C at 20°C/min and held at 310°C (5 min). Electron impact ionization was conducted at 70 eV, and detection was carried out in full scan mode with acquisition between 50 and 600 *m/z*. Compound identification was achieved using the NIST-2017 standard reference database and data comparison with compound standards. For sugar identification, the retention times using pure standards treated under identical conditions and assigning the same peaks of several isomers obtained after derivatization were used. The relative concentration values were calculated by dividing the peak area of the compound by the adonitol/ribitol peak area and the amount in milligrams of lyophilized material used.

For the detection of phenanthrene and of phenanthrene metabolites, lyophilized exudates from samples with clover plus phenanthrene after incubation for 3 days (8 mg) and for 6 days (28.2 mg) and lyophilized exudates from samples of clover plus phenanthrene plus strain HR1a after 3 days (20 mg) and after 6 days (46 mg) were extracted twice with 5 ml of hexane/acetone (1:1). After centrifugation, the samples were concentrated to dryness and dissolved in 1 ml of dichloromethane. Five microliters was injected as described above to detect phenanthrene/anthracene. For the analysis of the other metabolites, the rest of the volume was dried with N_2_ and derivatized (with the silylation reagent as above) at a final volume of 100 μl. Relative amounts of phenanthrene and salicylate (intermediate of the PAH degradation pathway and a plant metabolite) were given as peak area per milligram of exudates.

### Analyses of *pahAB* expression with different compounds present in the rhizosphere and in the gnotobiotic systems.

The analysis of *pahAB* expression was carried out by microtiter plate-based assays with the strain *Novosphingobium* sp. HR1a (pKSPA-1) (34) using Greiner black 96-well plates. The overnight cultures grown in LB were diluted (after washing three times with distilled water) in M9 minimal medium (74) supplemented with different carbon sources at a concentration of 10 mM (glucose, fructose, maltose, galactose, isoleucine, proline, glutamic, l-alanine, pyruvate, and *p*-hydroxybenzoate) to an OD_660_ of 0.05. Growth (30°C with continuous shaking at 360 rpm) and fluorescence (excited at 485 nm and read at 535 nm) were monitored in a Varioskan LUX multimode microplate reader (Thermo Fisher Scientific Inc., Singapore). The experiments were conducted in triplicate over 48 h, and measurements were taken every hour.

To analyze the *pahAB* gene expression in gnotobiotic systems, the glass jars, prepared as described above, were inoculated with the biosensor strain *Novosphingobium* sp. HR1a (pKSPA-1) at an initial OD_660_ of 0.1. The glass jars were incubated in the plant chamber under the same conditions as those described above. On days 3, 6, and 9, 200-μl samples were taken and deposited in a microtiter plate, and measurements were performed in the above-mentioned microplate reader.

### RNA-Seq experiments.

Overnight cultures grown in M9 minimal medium plus glucose of *Novosphingobium* sp. HR1a were diluted in the gnotobiotic systems prepared as described above to reach an initial OD_660_ of 0.1. The systems were placed in a growth chamber with the same conditions as those described above. Eight days after planting and inoculation, the liquid media of 4 pots were mixed and centrifuged (6,000 rpm during 10 min). The pellets containing the cells were immediately frozen in liquid N_2_ and stored at −80°C. Two independent experimental replicas were analyzed.

Total RNA was extracted using the TRIzol method (TRIzol RNA Isolation Reagents, Thermofisher Scientist), followed by further DNase treatment and purification with the RNeasy minikit (Qiagen). RNA degradation and contamination were monitored on 1% agarose gels. RNA purity was checked using the NanoPhotometer spectrophotometer (IMPLEN, CA, USA). RNA integrity and quantification were assessed using the RNA Nano 6000 assay kit of the Bioanalyzer 2100 system (Agilent Technologies, CA, USA).

One microgram of RNA per sample was used to prepare the sequencing libraries, which were generated by Novogen (Hong Kong) using NEBNext Ultra RNA library prep kit for Illumina (NEB, USA) following the manufacturer’s recommendations, and index codes were added to attribute sequences to each sample. The clustering of the index-coded samples was performed on a cBot cluster generation system using PE cluster kit cBot-HS (Illumina) according to the manufacturer’s instructions. After cluster generation, the library preparations were sequenced on an Illumina platform and paired-end reads were generated.

Raw data (raw reads) of FASTQ format were first processed through in-house scripts (Novogen, Hong Kong). In this step, clean data (clean reads) were obtained by removing reads containing adapters and reads in which uncertain nucleotides (N) were more than the 10% of the read length. Reads with low-quality nucleotides (base quality of <20) in more than 50% of the read length were also discarded.

Paired-end clean reads were mapped to the reference genome (JABXWS000000000) using HISAT2 software v.0.6.1. HISAT2 uses a large set of small GFM indexes that collectively cover the whole genome. These small indexes (called local indexes), combined with several alignment strategies, enable rapid and accurate alignment of sequencing reads. HTSeq software was used to count the read mapped to each gene, including known and novel genes. The RNA-Seq reads were assembled according to the reference genomes using Rockhopper (75) and then compared to known gene structures so that novel gene transcripts were predicted. The novel transcripts were aligned to sequences in the NCBI NR database using BLASTX (cutoff: e value of <1e−5). Novel transcripts with NR annotations were considered novel potential protein coding transcripts. In total, 1,038 putative novel genes were identified.

The level of gene expression was measured by transcript abundance. In our RNA-Seq analysis, the gene expression level is estimated by counting the reads that map to genes. Read count is proportional to the actual gene expression level but is also proportional to the gene length and the sequencing depth. To be comparable, the FPKM (fragments per kilobase of transcript sequence per millions of sequenced base pairs) is used. This method takes into account the effects of both sequencing depth and gene length on counting of fragments (76).

The quality of RNA reads and exploratory analyses of RNA-Seq samples are shown in Fig. S2.

10.1128/mSphere.00412-21.2 FIG S2 Quality of sequencing analysis of TH1 (experiment 1) and TH2 (experiment 2). (A) Violin distribution of samples. (B) Classification of raw reads and quality analysis. Download FIG S2, TIF file, 0.9 MB. Copyright © 2021 Molina et al. 2021 Molina et al. https://creativecommons.org/licenses/by/4.0/ This content is distributed under the terms of the Creative Commons Attribution 4.0 International license.

The levels of transcription (measured as FPKM) of well-known reference genes such as *dnaK* (chaperone), *recA* (recombinase A), *rpoD* (sigma-70 factor), *secA* (a component of the Sec protein translocase complex), *rho* (transcription termination factor), and *proC* (pyrroline-5-carboxylate reductase) (77–80) were taken into consideration for data analysis (Table S3). Genes with FPKM higher than that of RecA were arbitrarily considered highly expressed in the gnotobiotic systems.

### Statistical analyses.

Statistical analyses were carried out using SigmaPlot software via the *t* test (α = 0.05). The analyses of the bacterial persistence in nonsterile microcosms were carried out using the Mann-Whitney rank sum test.

### Data availability.

Read data were deposited in the Gene Expression Omnibus repository (GSE172273).
