# Supplementary figures and images for: Clover Root Exudates Favor Novosphingobium sp. HR1a Establishment in the Rhizosphere and Promote Phenanthrene Rhizoremediation
Source: mSphere. 2021 Aug 11;6(4):e00412-21. doi: 10.1128/mSphere.00412-21 (PMC8386446; doi:10.1128/mSphere.00412-21)

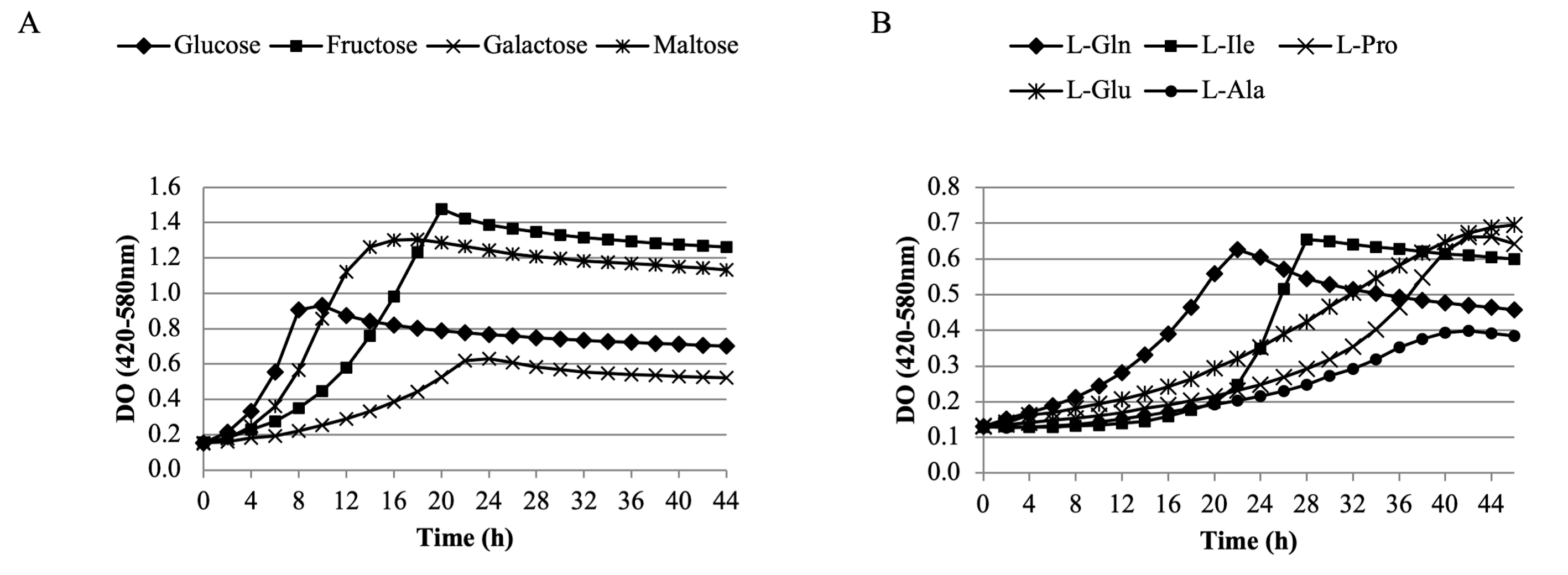

Supplement: FIG S1 [file msphere.00412-21-sf001.tif]

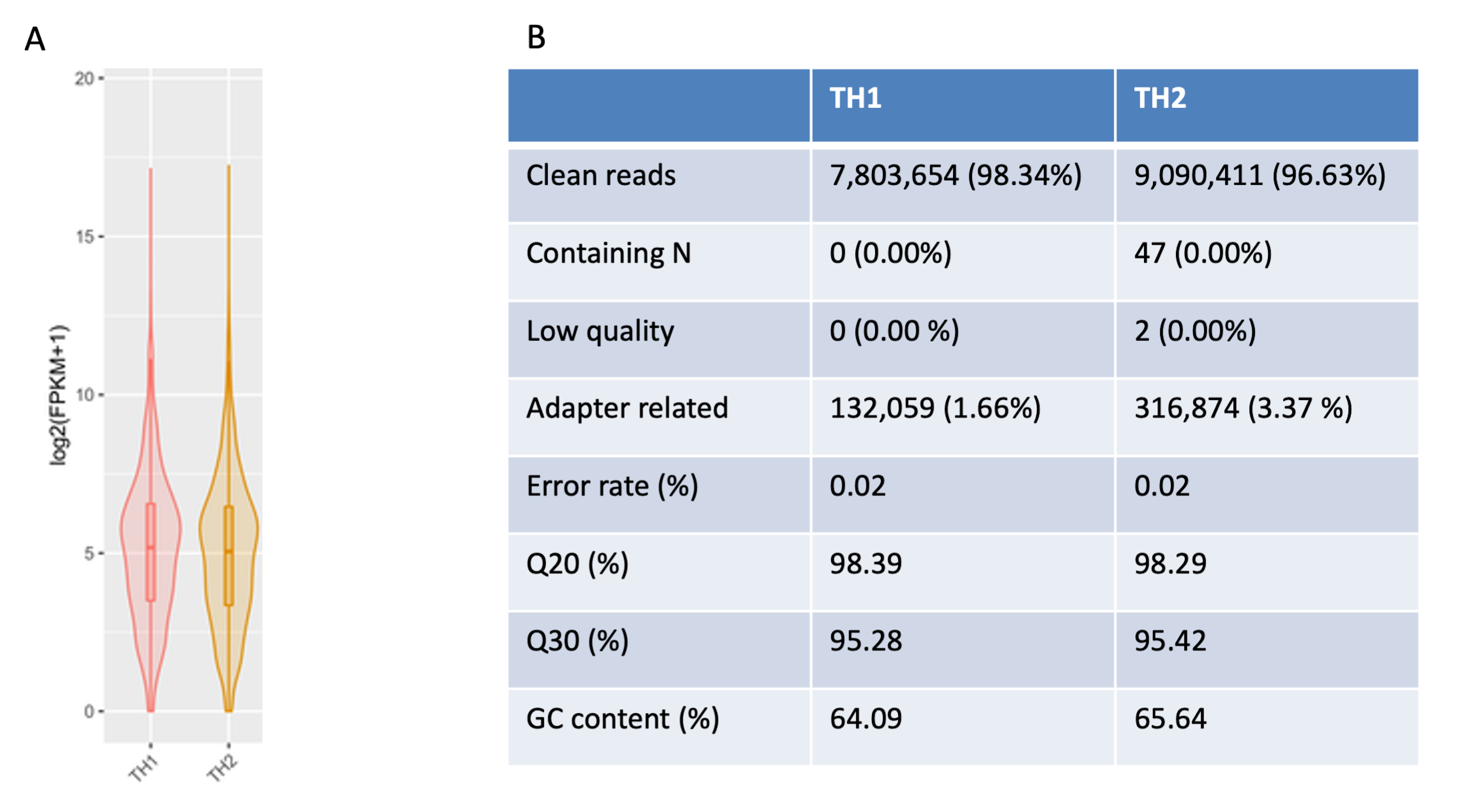

Supplement: FIG S2 [file msphere.00412-21-sf002.tif]
