# Supplementary material for: Clover Root Exudates Favor Novosphingobium sp. HR1a Establishment in the Rhizosphere and Promote Phenanthrene Rhizoremediation
Source: mSphere. 2021 Aug 11;6(4):e00412-21. doi: 10.1128/mSphere.00412-21 (PMC8386446; doi:10.1128/mSphere.00412-21)
Supplement: TABLE S2 [file msphere.00412-21-st002.docx]

**Table S2**: Identification of phenanthrene and salicylate in exudates. The relative amounts of each compound were calculated by dividing the peak area between the milligrams of lyophilized exudate used in the analysis. C: Clover, Phe: Phenanthrene, 3D: exudates obtained 3 days after inoculation, 6D: exudates obtained after 6 days.

|  | **Phenanthrene** | **Salicylic acid** |
| --- | --- | --- |
| C + Phe 3D | 2.15E+05 | 4.02E+02 |
| C + Phe 6D | 1.97E+05 | 7.04E+02 |
| C + HR1A + Phe 3D | 7.96E+03 | 2.56E+03 |
| C + HR1a + Phe 6D | 1.28E+03 | 4.39E+03 |
