# Supplementary material for: Clover Root Exudates Favor Novosphingobium sp. HR1a Establishment in the Rhizosphere and Promote Phenanthrene Rhizoremediation
Source: mSphere. 2021 Aug 11;6(4):e00412-21. doi: 10.1128/mSphere.00412-21 (PMC8386446; doi:10.1128/mSphere.00412-21)
Supplement: TABLE S3 [file msphere.00412-21-st003.docx]

**Table S3:** Level of expression (FPKM) of reference genes in *Novosphingobium* sp. HR1a growing in the gnotobiotic systems.

| Reference gene | Count-reads (fpkm) | Genebank ID | Number of genes with higher level of expression |
| --- | --- | --- | --- |
| *dnaK* | 91731 | HWN72_07395 | 12 |
| *rpoD* | 10153 | HWN72_06980 | 229 |
| *rho* | 8257 | HWN72_1644 | 295 |
| *secA* | 7606 | HWN72_05855 | 319 |
| *recA* | 5581 | HWN72_10800 | 417 |
| *proC* | 897 | HWN72_12565 | 1915 |
